# Projecting Spanish fertility at regional level: A hierarchical Bayesian approach

**DOI:** 10.1371/journal.pone.0275492

**Published:** 2022-10-18

**Authors:** José Rafael Caro-Barrera, María de los Baños García-Moreno García, Manuel Pérez-Priego

**Affiliations:** Department of Statistics and Econometrics, University of Córdoba, Córdoba, Spain; University of Almeria, SPAIN

## Abstract

The transition from a demographic regime of high mortality and high fertility to one with low mortality and low fertility is universal and comes along with the process of socio-economic modernization. The Spanish total fertility rate has decreased to below replacement levels in the last decades. The decline has persisted since the 1960s and is diverse across the country. Based on that diversity, the use of population forecasts, not only at national but at regional levels, for planning purposes (governments and private sector) with large horizons has become a must to provide essential services. Using a Bayesian hierarchical model we constructed probabilistic fertility forecasts for Spain at the regional level. Although this approach is already issued by the United Nations little research has been done focusing on the Spanish subnational level. Our objective is to disaggregate the national projections of the total fertility rate for Spain into regional forecasts. The results of this research will show the model fitting, first to the national level and then using a multifaceted and continuous evolution of fertility over time, at the regional level, to check its convergence.

## 1 Introduction

While increasing life expectancy is a symptom of social progress, it is also a challenge for governments, private pension schemes and life insurers because of its impact on health care costs and pensions. A large number of demographic and actuarial studies have recognised the problems caused by ageing populations, low fertility rates and increasing longevity, therefore attention has focused on the development of methods for forecasting and projecting fertility and mortality rates [1–4].

The decline in fertility has been accompanied, in developed countries, by major changes affecting the role of women and the family. Some authors speak of a *second demographic transition* [5–8], although for some others [9–11], the term *gender transition* would be more appropriate. In fact, according to the latter, the main difference concerning the first demographic transition is that a cultural component has been added. In effect, these changes are linked to the new role of women in society and its presence in the labour market and to the transformation of the organization of demographic reproduction, traditionally based on a clear separation between women, who are confined within the family, and men, who enter the labour market. With the progressive presence of women in that market, the articulation between family and work has become a central issue of our time. In this context, [12] analyses the female labour force participation in developing countries, finding that it affects to economic activity, educational attainment, fertility rates and social norms. Also, [13], study the determinants of labour force participation of urban married women in eight low- and middle-income economies to understand what drives changes and differences in participation rates. Also in this context but focusing on a specific country, [14], discuss the cultural change and female labour market participation in Germany, analyzing the effects of family policy on fertility.

Europe is one of the continents where the demographic problem mentioned above is highlighted. The TFR (total fertility rate) is the average number of children born to women during their reproductive years) and in most of the EU member states, is below the replacement threshold of 2.0–2.1 births per woman [15].

The fertility replacement threshold is the TFR a nation needs to stabilize the population. In a scenario where the TFR falls below 2.0–2.1 births, as the population ages and declines, economic growth and funding for government programmes are reduced, fuelled by fewer employees available to work, pay taxes and fund government social security programmes [16, 17].

**Fig 1:**
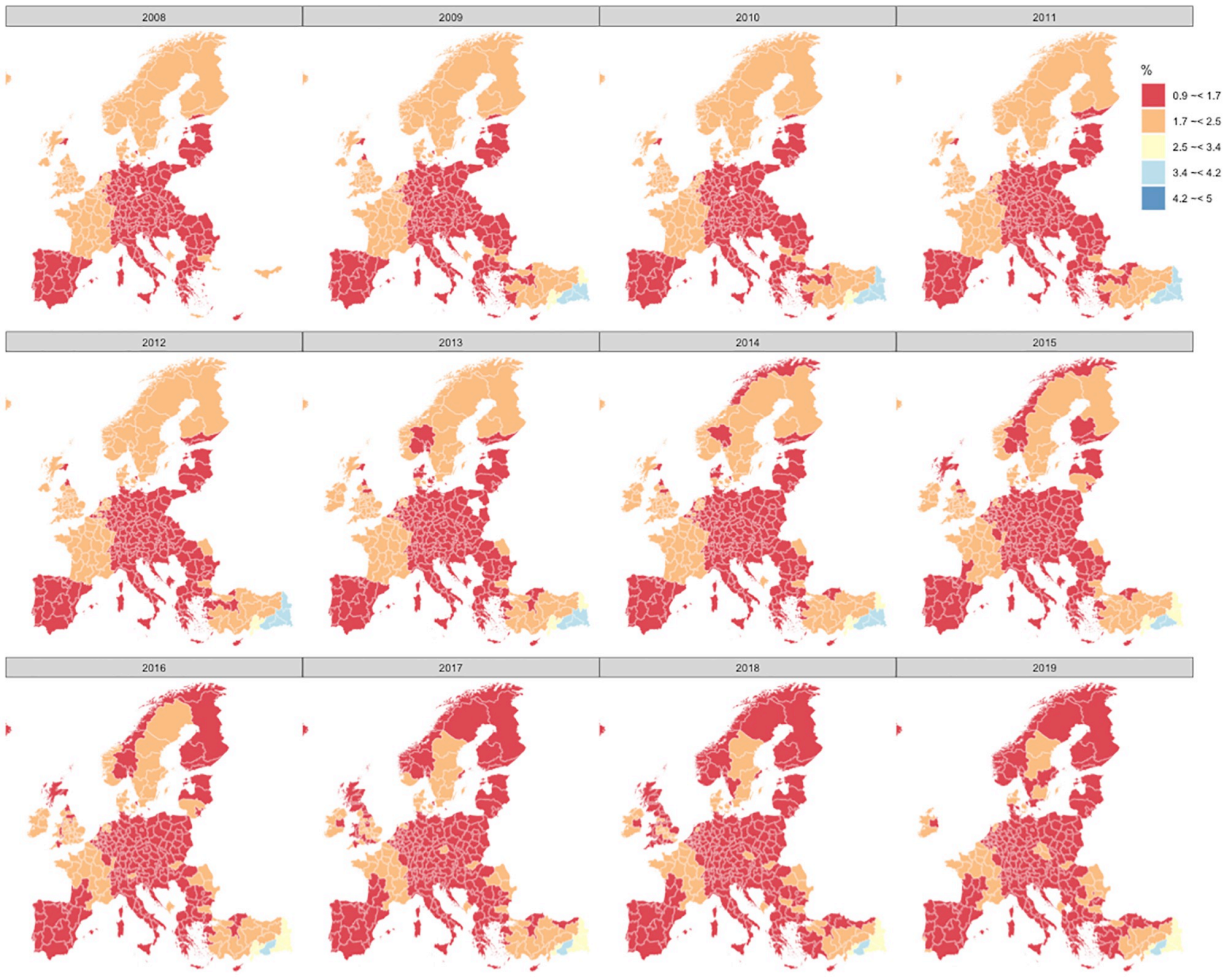
TFR at NUTS-2 level. Average number of live births per woman in Europe from 2008 to 2019. *Source:* Data from Eurostat and map built with the open source software R and package ‘Eurostat’.

Fig 1 shows the evolution of the TFR rate in Europe at NUTS-2 level (NUTS stands for *Nomenclature of Territorial Units for Statistics* and is the UE harmonised manner of collecting data in the different geographical areas) from 2008 to 2019 where it can be seen to be declining to below the replacement threshold in most countries [18].

The situation in Spain is similar to other European countries and this behaviour of the fertility rate has been accentuated during periods of financial and economic crisis. The increase in unemployment levels, job uncertainty and, in general, the economic difficulties for families, together with the decrease in marriages, have delayed or postponed, in many cases, the first childbearing. This topic is extensively developed by [19] who have conducted much research on the connection between uncertainty and fertility.

**Fig 2:**
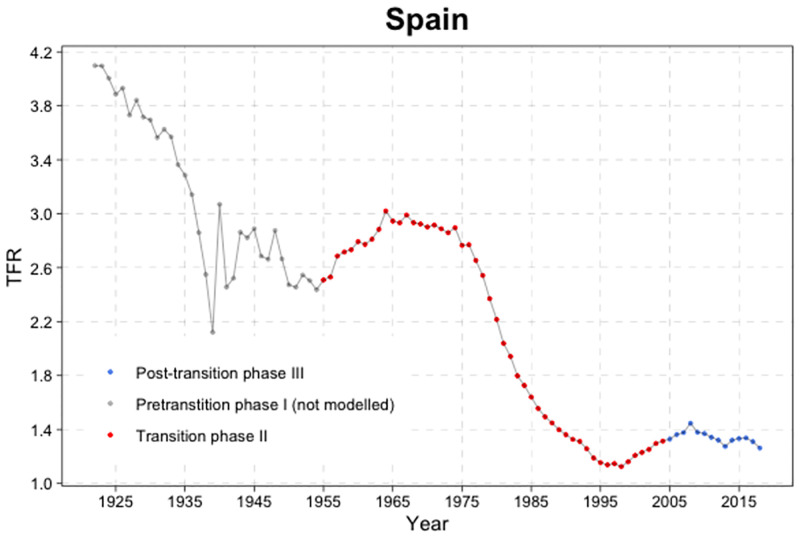
Number of births in Spain from 1960 to 2020. *Source:* Data from the Spanish Statistical Office (INE).

Fig 2 shows the number of births in Spain from 1960 to 2020. From 1999 the TFR and the number of births increased for 10 years, up to 2008, after both indicators fell dramatically, in fact, 2020 registered the lowest number of births in Spain in the last 60 years, with just under 340.000 births.

**Fig 3:**
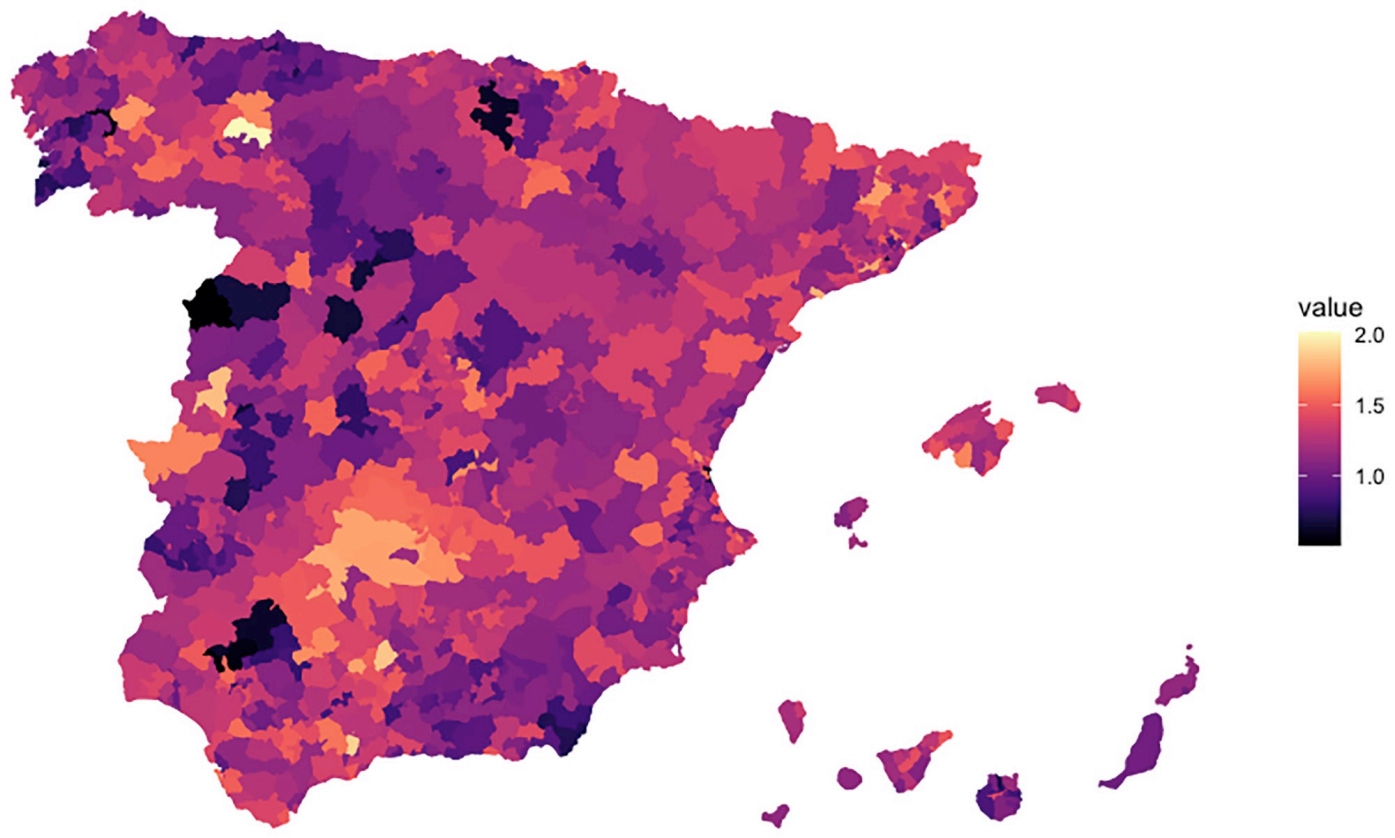
TFR in 2018 at municipality level. *Source:* Map generated with R software. Data from the Spanish Statistical Office (INE) and shapefiles for the map from www.gadm.org.

However, the socio-economic differences within Spain and the geographical diversity between the north and south make a demographic analysis at the regional level necessary to verify TFR convergence, in this sense, [20, 21] have studied Spanish fertility using regional or provincial fertility rates. More recently, [22] analyze fertility variation by showing the geo-spatial distribution of fertility across regional geographical areas in Spain between 1981 and 2018. For instance, Fig 3 shows the Spanish TFR at the municipality level. This level shows that in most municipalities the TFR is lower than 2.0 and especially remarkable are those with a TFR close to 0. As the size of the municipalities has an impact on birth rates, the characteristics of these barren territories where TFR is almost 0, are usually quite similar: inland, sparsely populated and ageing [23–26]. Therefore, fertility differences are related to two linked factors: the degree to which a region (or municipality) is influenced by the age of the population and the age structure contrasts between capital cities and the rest of the province’s regions. As the standard age range for the TFR is aged 15–49, an older region will have an impact on the TFR. These two variables make a division between, on the one side, the foremost energetic and more youthful areas and the drained, elderly and demographically stagnant territories (cities like Huesca, Cuenca, Soria, Ávila, Ourense,…, for instance), on the other side.

**Fig 4:**
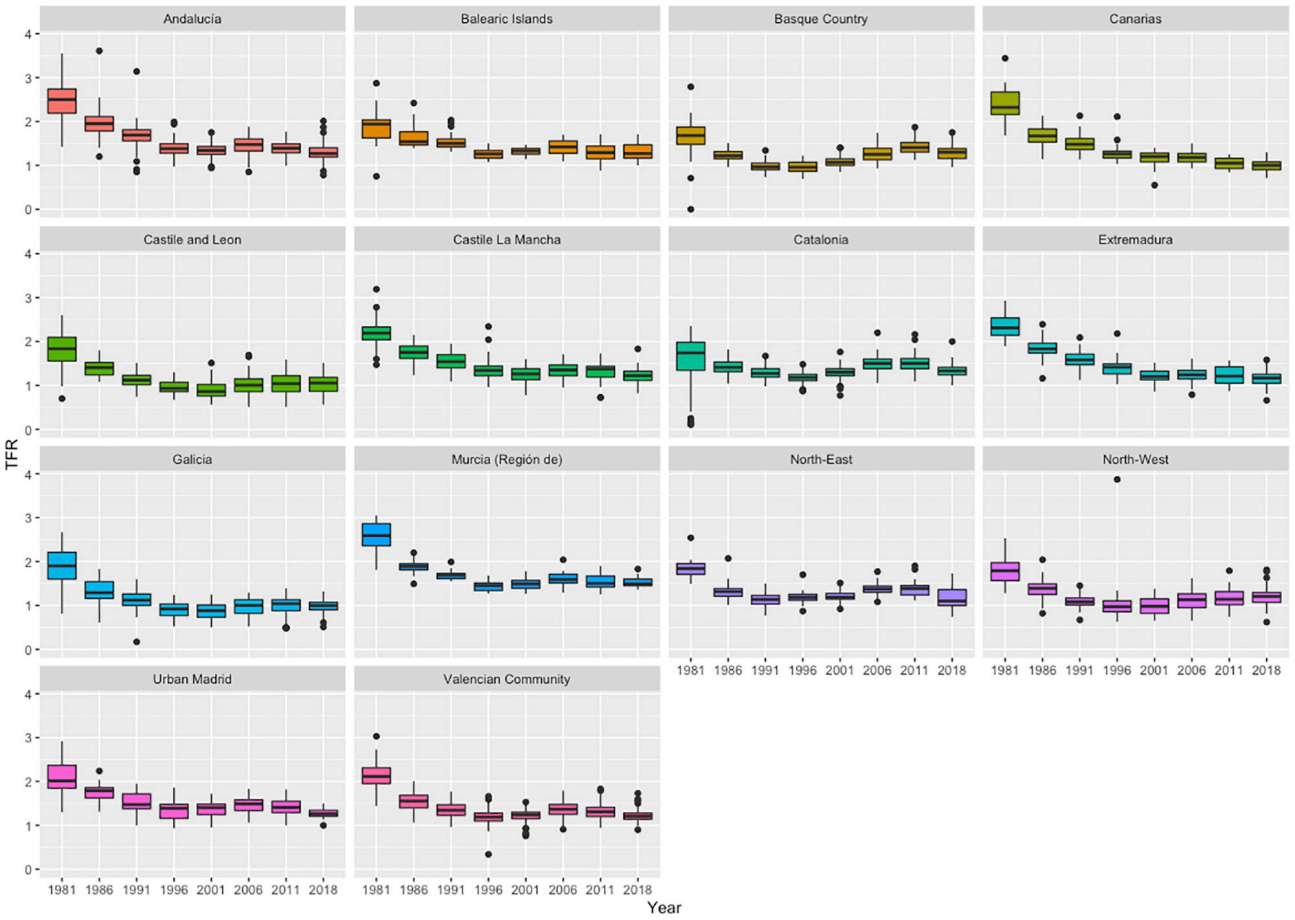
TFR evolution from 1981 to 2018 by region. *Source:* Data from the Spanish Statistical Office (INE).

Fig 4 on the contrary shows the evolution of the TFR from 1981 to 2018 by region.

Nevertheless, very few sub-national projections of fertility in Spain, at the regional level, have been made using the methodology we propose, so in this paper, we aim to project the Spanish TFR at a regional level by adopting the approach proposed by [27], using a Bayesian hierarchical model. The rest of the work is organized as follows: we begin by reviewing the existing literature on fertility models. The next section is focused on the methodology adopted and the data we will work with. The results section shows the model fitting, first to the Spanish population as a whole and then using a multifaceted and continuous evolution of the TFR over time, at the regional level, to check its convergence, in addition to that, we will discuss the main differences between regions. Finally, we will conclude with the main demographical aspects to take into account when modelling Spanish fertility and discuss the drawbacks of probabilistic models.

## 2 Background: A literature review on fertility and age structure models

Modelling fertility curves has been of interest to demographers for a long time so through a variety of mathematical models, it has been possible to describe age-specific fertility patterns with specific shapes in all human populations over the years. Even though the demographic literature is offering a wide variety of fertility measures, the TFR is a key indicator for population projections due to its simplicity, ease of interpretation and availability. For instance, the United Nations Population Division produces population projections based on this measure [28, 29]. However, the adoption of the TFR as a standard measure for population projections is not new, for instance, [30] pointed out that this indicator had been already used by the U. S. Census Bureau. On the other hand, [31], conduct analyses on the relationship of low and lowest-low period fertility to cohort fertility and key fertility-related behaviour using the TFR as the main indicator. Also, [32], provide a thorough review of the literature on fertility research by classifying existing studies according to the determinants of fertility. However, according to Bongaarts and Feeney (1998), [33], fertility forecasting based on TFR has limited success because of the inefficiencies in this fertility indicator. Based on this, the authors, introduce two components or effects at the time of estimating fertility, arguing that changes in fertility can occur because of these two effects: the *quantum* and the *tempo* effect. The *quantum* component refers to the TFR that would have been observed in the absence of changes in the timing of childbearing during the period in which the TFR is measured whereas the *tempo* effects are distortions due to changes in the timing of birth. [14] used the Bongaarts-Feeney framework as the theoretical backbone of their PC-based age-specific fertility prediction model, which was then aggregated into TFR predictions. The authors even go a step further and also discuss incorporating fertility strategies into fertility projections in low-fertility countries.

Despite the limitations of the TFR mentioned above, addressed by Bongaarts and Feeney, [33], the choice of this indicator in our research is because as we explained is one of the most widely used fertility measures as it provides an understandable measure of hypothetical completed fertility (TFR only includes live births) and it is unaffected by differences or changes in the age-sex composition. The fact that the model used for our predictions employs data from the United Nations and the Spanish INE, both use the TFR as a basic indicator.

In the remainder section, we propose a literature review on approaches that explain fertility modelling combined with works that model the age structure, we think this relationship is important because an imbalance in age structures creates important problems at institutional stability levels (e.g. schools), labour force succession or old-age social security. Shrinking class sizes, workforce shortages and payroll tax collections that are insufficient to pay for retirement benefits are all consequences of long periods of below-replacement fertility.

In 1956, Kingsley Davis and Judith Blake, developed the *intermediate variables model of fertility*. The intermediate variables analytical system consists of the disaggregation of the process by which a person is born, which revolves around three key moments: (1) intercourse, (2) conception and (3) gestation and childbirth, giving rise to eleven fertility-related variables [34]. Such a system was revised by J. Bongaarts and R. J. Potter in 1983, turning it into the model of *proximate variables*. The proximate variables proposed by Bongaarts are based on three biological variables: (1) the postpartum barren interval, (2) the fertile interval and (3) the time to full pregnancy (assuming a constant duration of 9 months) [35]. According to the authors, these variables are the only ones susceptible to being affected by socio-economic and environmental factors (according to Bongaarts) or cultural factors (according to Davis and Blake). Aguinaga’s application of Bongaarts’ model of fertility to the Spanish case is interesting from the point of view of the contrast between the Natural and Social Sciences, since this model, according to the author, is too ‘biological’ and too little ‘sociological’ and does not show any causal relationship between the two realities, the biological and the causal [36].

Some of these fertility models mentioned above are quite effective in fitting the fertility rate distributions, for instance, [37], using the least squares technique adjusted the fertility curve for age-specific rates for a decade (from 1962 to 1971) in Denmark. Among the functions fitted were: a cubic spline, the Hadwiger and Coale-Trussell functions, the gamma and beta densities, two versions of a polynomial, and two of Brass’s relational procedures, as well as the Gompertz curve. Results showed that the spline function fitted all curves better than any of the others. The Coale-Trussell procedure and gamma density were about equal, followed by the Hadwiger function and fit the data well. One of the polynomials fit reasonably well, but the rest of the functions were less accurate.

However, since Lee and Carter proposed their well-known model for forecasting mortality, [38], a family of demographic models based on it has arisen. Hence, the Lee-Carter (LC) approach for forecasting age patterns makes the model suitable for modelling fertility as well. For instance, [39] develops methods for using time-series techniques to make constrained long-term forecasts of fertility where the principal interest is in the variance and the autocorrelation structure of the forecast errors. On the other hand, [40], compares and assesses variants of the curve fitting and principal components techniques (Lee-Carter model among them), to project age-specific fertility rates. Also based on the Lee-Carter model, [41] proposed a method for forecasting age-specific mortality and fertility rates observed over time by applying a combination of functional data analysis, nonparametric smoothing and robust statistics.

Nevertheless, difficulties arise when an attempt is made to ascertain the causes of changes in fertility from an economic point of view (beyond quantifiable factors), in this sense, family decisions in a socio-economic and psychological framework play a key role in fertility. The economic analysis of fertility formulated by Becker in the ‘60s is important to understand this demographic variable within an economic framework and it deserves to be explained in more detail, [42]. Becker’s contribution in this field is decisive and comes at a time known as the *“demographic transition”*, i.e. the transition from a sharp decline in the birth rate that took place during the 1930s to a sharp rise in the birth rate in the post-war period. In this period, in 1960, the publication date of his pioneering work on economics and fertility, Becker constructed a theory in which a family (or couple if they had not yet children), measured the utility of their consumption by including the number of surviving children. In this way, he presented children as durable goods because they provided *“psychic satisfaction”* to their parents over a long period, he even argued that they could be durable goods of production in certain contexts and showed that *“the theory of demand for durable consumer goods is a useful framework for analysing the demand for children”*.

Using this Economic Theory of Demand, Becker adapts it and builds his *Economic Theory of Demand for Fertility Decisions* (this name does not exist actually as Becker did not formulate any theory of demand for fertility ‘per se’, we just simply used the name to distinguish it). In it, he argues that like other durable goods, children also provide “utility” and this utility is measurable and comparable to that of other goods through their corresponding utility function or set of indifference curves. In this sense, preference for certain tastes allows for differences in fertility.

The **quality** of children is also an aspect that influences the *demand* for them, in the sense that a family should not only determine the number it wants to have but also the amount it spends on them, establishing the concept of “superior quality” for those children on which parents spend more money.

The level of **income** is another factor that influences the “quality” of children since it can be expected that an increase in the level of income, in the long run, should increase the amount spent on each child and consequently an increase in the quality of the children.

On the other hand, Becker argues that the net **cost** of children can be calculated as either positive or negative. If they were positive, the children would be a durable consumers as a whole and it would be necessary to assume that an income or psychic utility is received from them. If the net costs were negative, the children would be a durable good of production and a pecuniary rent would be received from them.

Finally, the concept of **supply** in this *Economic Theory of Demand for Fertility Decisions* is subject to two important aspects: on the one hand, a certain uncertainty that implies distinguishing between current utility and expected utility, and on the other hand, the number of children is not only related to the level of income and prices, but also to the ability to have children. In the 1960s, the average number of live births produced by married women in societies with little knowledge of contraception was very high, so the average family was more likely to have a high number of children than a low number.

Subsequently, as knowledge of contraceptive methods became more popular, the quality of children and the number of children decreased.

The main conclusion and what we can extract from Becker is that family decisions, and above all when it comes to having children, are taken in a social and economic environment in which the quantity and quality of children are closely related and depend on socio-economic variables and factors. Thus, an increase in income or a decrease in the cost of children would affect both the quantity and quality of children, usually increasing both. The quality of children is very important in its own right, as it determines the education, health and motivation of the future workforce.

In 1988, along with Barro, Becker extended his theory arguing that altruistic parents choose fertility and consumption by maximizing a dynastic utility function [43]. In their work, Becker and Barro developed an economic analysis that implies several linkages: first, between fertility rates and capital accumulation across generations. The utility of parents depends not only on their own consumption but also on the utility of each child and the number of children. By relating the utility of children to their own consumption and to the utility of their children, the authors obtain a *dynastic* utility function that depends on the consumption and number of descendants in all generations. To maximize this *dynastic* utility function, according to the authors, the first-order conditions imply that any generation depends positively on the real interest rate and the degree of altruism. The second linkage is between the effects on fertility of mortality of children, subsidies to (or taxes on) children and social security and other transfer payments to adults. The third connection links fertility and population growth to international capital markets showing that fertility in an open economy depends positively on the long-term interest rate and negatively on the rate of technological progress.

In the same line, that is, the one explaining there are other components influencing fertility, [44] concludes that the economic perspective is insufficient for this task and that some other factors or contexts play an important role, such as cultural and social.

Subsequently, in models built in recent years, the patterns of fertility data in developed countries show considerable variation related to the fertility curve. In this sense, [45] proposed a model where flexibility is the key aspect because it combines the old and new patterns of fertility and gathers the influence of the practices in marriage and childbearing, remarriage, widowhood and divorce.

Countries show inequalities concerning the age at which fertility rates peak and the varying speed with which the peak is approached from the beginning and then passed to reach the end of the fertility period, [46], other countries such as the United Kingdom, Ireland, and the United States show heterogeneous fertility patterns, which may be associated to some extent with marital status and the educational and social status of mothers. Moreover, in these countries, this heterogeneity in fertility patterns can be explained by ethnic differences in the timing and number of births [47, 48].

Because of this heterogeneity, existing models cannot capture the modern fertility pattern. To overcome this, [49] propose a mixture of Hadwiger’s function. The Hadwiger function is given by the expression $h\left(x\right)=\frac{\alpha \beta }{\gamma \sqrt{\pi }}{\left(\frac{\gamma }{x}\right)}^{\frac{3}{2}}exp\{-\beta^{2}(\frac{\gamma }{x}+\frac{x}{\gamma }-2)\}$, where *α*, *β* and *γ* represent the estimated parameters and *x* is the age of the mother at the birth of the child.

Among the models used to represent the age-specific fertility pattern of populations that do not show high early fertility, several have been shown to provide ‘fairly’ accurate fits to fertility distributions. These include, for example, the parametric fertility model proposed by [50, 51], which is still widely used today; it is a hybrid model (empirical and parametric) and its basic form is

$$f\left(a\right)=T\cdot G\left(a\right)\cdot n\left(a\right)\cdot e^{m\cdot v(a)}$$

It is therefore a four-parameter fertility model *(m, M, a_0_* and *k)*, which are more parameters than necessary for efficient representation. Although the model has the virtue of having parameters that have some real-world interpretations, these parameters cannot be measured in real populations, so it is difficult to fit the model to them. Extensions to the Coale-Trussell model have been proposed by [52–54] to make it less restrictive and more capable of capturing fertility in a wide range of populations, although the suggested modifications are relatively minor in terms of their overall impact.

The Beta and Gamma distributions are equivalent to Pearson’s Type I and III curves, respectively, proposed by [37], the same Hadwiger distribution (also mentioned above) and the cubic *splines* [55, 56] has also been found to be highly accurate and are in common use. In addition, the Pearson Type I curve [57], Pearson’s Type III Curve [58] and the model proposed by [59] have shown a good predictive fit, since all of them, directly or indirectly, have their origin in the P/F ratio method proposed by William Brass in 1964, which, although it has been modified and replaced, in its original form, by Gompertz’s relational model, the basis of the method is based on the observation that if fertility has been constant over a long period, the cohort and period fertility measures will be identical. Under constant fertility conditions, the cumulative fertility of a cohort of women up to any given age will be the same as the cumulative fertility up to that same age in any given period. If we assume that there are no appreciable mortality differences in maternal fertility, so that surviving women do not have significantly different levels of childbearing than deceased women, the cumulative fertility of a cohort of women up to a given age is the same as the *Mean Parity* in that cohort (this assumption is not very important, since even if there are differences in the fertility of living and deceased women, in most populations, the magnitude of female mortality at reproductive ages is very small and therefore the differential survival effect will be small). Brass defined *P* as *“the average parity (cumulative lifetime fertility) of a cohort of women up to a given age, and F is closely related to the cumulative fertility (period) up to that same age”*. The ratio method expresses these two quantities about each other in the form of a ratio for each age group [60].

The main weakness of the method is that in reality, the data are never free of error, so the hypothesized pattern of deviation from the unit ratio *P/F* is confounded by the underlying errors in the data.

The second type of error encountered, less frequent nowadays in developed countries as births are reported by the hospitals and will not encounter these problems with this reporting system, is that women tend to omit some of their live-born children, particularly those living in other households and those who have died, with the result that the proportion omitted tends to increase with the age of the mother, causing the *P/F* ratio to increase [28].

To overcome this, Gompertz’s relational model is presented as a versatile improvement since it uses the same input data (and makes the same assumptions about errors affecting fertility data) as its precursor. However, it is important to remark that the method does not require the assumption that fertility has been constant in the past [61, 62].

Several other authors focus their analyses on applying and fitting the models described above to specific populations and specific countries, for example, [63] use descriptive aggregate analyses to examine the relationship between the low (and the lowest) period of fertility and the cohort fertility and the key fertility-related behaviours, such as leaving the parental home, marriage and female labour force participation, in Europe from 1975 to 1999.

On the other hand, [64] approaches the issue from the perspective of developments in Israel and Palestine by discussing some possible future scenarios for the emerging population, highlighting the variety of demographic and social processes that have affected or may affect the change in size and distributions of the population of Arabs and Jews in the territory between the Mediterranean Sea and the Jordan River.

Instead, [65] applies the ‘variable-*r*’ method (variable-*r* means ‘variable rate’) from the Preston and Coale model. This model is the second of what later became known as the *Death Distribution Methods*, for estimating the completeness of death reporting about a population estimate at a given point in time, although primarily used for mortality projections, the variable rate approach of Preston and Coale means that it can be used to estimate various demographic measures. Therefore and based on that method to assess the level of fertility in China using census data as well as annual population surveys from 1990 to 2000, concluded that, with the proposed methodology, fertility in the country has reached a level well below replacement level.

In the American continent, [66] presents Brazilian fertility trends during the previous (20th) and present (21st) centuries and, emphasizes the importance of individual profiles for fertility decisions and [67] examines the socioeconomic associated with cumulative fertility in Ghana. Negative binomial regression models were used to estimate determinants of cumulative fertility using data from the Ghana Demographic and Health Surveys of 2003, 2008, and 2014

If we turn out our attention to Europe, interesting is also the approach of [68], as they apply functional data models and time-series methods to forecast the components of change in mortality, fertility and net international migration and use them in forecasting the population of France [69]. Such a probabilistic population forecast is compared with the official population projections for the country, which are based on traditional deterministic scenarios. For France, also [70] propose a Spatio-temporal geostatistical model for the TFR of the country whose objective is the simultaneous study of the spatial and temporal behaviour of the TFR for predictive purposes.

However, [71], focus their attention on Italy; in the former’s study, the aim is to describe the process of birth postponement and recovery in Italy, a country, like Spain, with persistently very low fertility levels, where they find that recovery is currently underway in the northern regions of Italy, signs of recovery that are, above all, evident among younger generations and more educated women. Still in Italy but more recently, [72], propose a dynamic model to describe and predict the evolution of Italian fertility rates for a specific age over time; In particular, they slightly modify the Gamma function to include stochastic time-varying parameters to describe the systematic and macroscopic variations of age-specific fertility rates over time, while a non-parametric geostatistical model is applied to describe the correlated residuals at the microscopic level.

On the contrary, [73] provides a new approach consisting of mixing several distributions to predict future women’s reproductive behaviour. In other words, starting from the most used distributions to studying the fertility curves, the Gamma distribution, the Beta distribution, and the Hadwiger function, the author has tried to approximate the method for Estonian data.

Perhaps, one of the most interesting works in the last years is the one by Burkimsher who studies the evolution of the fertility curves of the 1968 to 1980 cohorts of women in 22 developed countries showing that for some countries the transition from an early to a late fertility schedule goes through a phase when the first birth fertility curve is bimodal. In other countries, a model ‘shoulder’ is apparent and concludes that the existence of a bimodal fertility curve suggests the polarization of women into one group that remains longer with an early fertility schedule and a second group that moves more rapidly on to a later schedule [74].

For the Spanish case, [75], offers a new proposal. In the period between 1985 and 1999, the author studies whether the significance of religion on fertility (both in family size and in the birth interval) has changed during that time.

On the other hand, [76], propose a parametric model for fitting fertility curves based on a combination of two Weibull functions, which performs a good fit in countries where the fertility curve shows a non-traditional pattern, however, is also suitable for more ‘classical’ fertility curve fitting. The model depends on six parameters and is primarily designed to fit fertility patterns in countries with an early ‘age hump’. Based on that, the authors conclude that while it does not provide the best fit in all simulated cases, it can improve the fit other models provide, depending on more parameters.

More recently and based on a more quantitative and technical approach, [77] present a methodology for generating stochastic population projections that combine the cohort-component method (the reference method used in most statistical studies in this area) with Monte Carlo simulation of two of the main demographic data: fertility rates (by age) and survival probabilities (by age and gender). The Monte Carlo simulation is based on a parameterisation of the corresponding curves and a multivariate time series model which is used to simulate future scenarios. In the part referring to fertility projections, the authors conclude that this projection for Spain will converge with that of the rest of the European countries also simulated, which is around 1.8 children per woman.

As stated before, extensions to the LC model combined with other approaches have been widely used in demographic models within a Bayesian framework have been developed. These models work better only when incorporating information about observed data or future patterns [78]. In this line, the work of [79] introduces a Bayesian projection model to produce country-specific projections of the TFR and uses a Bayesian hierarchical model to project future TFR based on both the country’s TFR history and the pattern of the countries. As it serves as the base model for our regional projections we will go deeper in the next section. Following this kind of approach, [80] proposes a Bayesian model for fertility that incorporates a priori information about patterns over age and time; also, [81] develop a dynamic Bayesian approach to forecasting populations by age and sex which embeds the LC-type models for forecasting the age patterns. Also, [82, 83] adopted a Bayesian hierarchical time series model to estimate and project the provincial sex ratio at birth.

Lastly, the pandemic has also affected the behaviour of the population as it brings socio-economic uncertainties that influence childrearing decisions. Although fertility intentions and behaviour are not identical and often do not run in parallel, is beyond any doubt that the Covid-19 pandemic has affected family dynamics. Moreover, the lack of data does not draw significant conclusions yet on the long-term effects of the pandemic on fertility. However, it is worth looking into the latest research on this subject as, like any other shock (economic crisis, outbreak of disease, natural disasters, wars,…) the impact on society; therefore, demography is determinant.

For instance, [84] assess the impact of the COVID pandemic on fertility intentions and behaviour in the Republic of Moldova, a middle-income country in Eastern Europe, using the Generations and Gender Survey. The contribution of [85] is interesting too as their study presents an overview of changes in fertility plans during the pandemic crisis in a sample of the population between 18 and 34 years old in Italy, Germany, France, Spain and the United Kingdom, showing that these plans were negatively revised in all countries although not in the same way.

On the other hand, [86], studied the most recent data on monthly births trends to analyze the initial fertility responses to the outbreak of the COVID-19 pandemic in 22 countries, associating this outbreak with an accelerated decline in the number of births in most of the highly developed countries studied in November 2020-January 2021. In the same line of work, [87] compiled the most recent available data for reported COVID-19 cases (deaths) worldwide, for 22 high-income countries, by comparing the disease profile to pandemics of the past, they assess its association with births, to try to understand how pandemics change population dynamics.

The contribution of [88] is equally interesting as their contribution tries to understand also the consequences of the Covid-19 pandemic on subnational fertility patterns in contexts of high inequality and limited governmental response, like Brazil and Colombia, which is relevant for other societies where geography and socioeconomic status are critical axes of inequality.

[89] aimed to contribute to the empirical literature by examining the effects of pandemics-related uncertainty on fertility behaviour. Indeed, they focus on precautionary savings motivation to explain the level of fertility rate and changes. Using a novel measure of pandemics-related uncertainty, the World Pandemics Uncertainty Index (WPUI), the authors show that uncertainty related to COVID-19 decreases the fertility rate.

Finally and despite what was mentioned earlier the lack of data does not yet allow us to assess the demographic impact of the pandemic but some works already try to project the future by incorporating the effects of COVID-19. For this reason, [90], in the absence of more timely data, use data from Google search term volume for keywords related to fertility to predict some features of fertility change expected from the pandemic in the United States at the state level that is: the direction, magnitude, and timing of fertility change. Also, [91] examine fertility trends up to 2019 in the United Kingdom and discuss the possible impact of the COVID-19 pandemic on childbearing behaviour. Based on this, the authors draw several possible future scenarios for fertility rates. On the other hand, [92] assesses the potential impact of the pandemic on fertility based on an adjusted version of a stochastic approach already used but for excess mortality estimation [93].

## 3 Methodology

### 3.1 Data processing

The data for Spain were obtained from registers at the municipality level, the *padrones municipales* (Municipal Population Registers). This population registers records individuals by recording their nationality, place of birth, age, or marital status. Since 1996, the Spanish *Padrón* has been updated annually with the National Statistical Institute (INE) being responsible for quality control. The INE makes this data available under the name *Padrón Continuo* (continuous register) and usually took 2 years for the Spanish population registers to become reliable.

To address the mapping of the TFR in Spain, in Fig 3, we have compiled data from the *Padrones municipales* on the INE website for 2018 (http://www.ine.es). Maps are based on the global administrative area database provided freely available for academic use (https://gadm.org/license.html), transformed by the authors to be consistent with INE data.

Evolution of the TFR, Fig 4 was built from 1981 to 2019 and for the projections of the TFR at the national and regional level, (Figs 6 and 7), data range from 1950 to 2018 with a 5-year periodicity and estimations are projected up to 2100. All the figures and projections were produced with the statistical software R and the research is fully reproducible with the files available in a public repository (see Data availability statement).

### 3.2 Methodology

As stated in the previous section, the proposed Bayesian model for fertility projection is based on [27, 79], therefore, ours is a derivation of the UN’s model to the sub-national level. In the model, a random walk with drift is used to project the TFR during the fertility transition, using a Bayesian hierarchical model to estimate the parameters of the drift term. The TFR is modelled with a first-order autoregressive process during the post-transition phase. It uses 5-year estimates of the TFR from 1950–1955 to 2005–2010 and is based on the observation that the evolution of the TFR includes three broad phases, referred to as, *Phase I*: a pre-transitional high fertility phase; *Phase II*: the fertility transition in which the TFR declines from high fertility levels towards or below replacement fertility level; and *Phase III*, a post-transition phase of low fertility, which includes the recovery from below-replacement fertility to replacement fertility and oscillations around fertility at that same level. The observation period for each region is divided into these different phases based on deterministic definitions of their start and end periods and then modelled separately. Thus, we define *τ*_*c*_ as the beginning of the *Phase II* for country *c*, which is given by

$$\tau_{c}=\{\begin{array}{c} \text{max}\left\{t:\left(M_{c}-L_{c,t}\right)<0.5\right\},\;\text{if}\;L_{c,t}>5.5\\ <1950-1955,\;\text{otherwise} \end{array}$$

where *M*_*c*_ is the result of the maximum observed TFR in region *c*, and *L*_*c*,*t*_ indicates the local maximum. The period of onset of Phase III, denoted by λ_*c*_ for region *c*, is observed within the observation period if two subsequent increases below a TFR of 2 have been observed. For these regions,

$$\lambda_{c}=\text{min}\left\{t:f_{c,t}>f_{c,t-1},f_{c,t+1}>f_{c,t}\;\text{y}\;f_{c,p}<2\;\text{for}\;p=t-1,t,t+1\right\}$$

is the TFR in region *c* and in period *t*. For the rest of the regions, *f*_*c*_ > 2005−2010

The proposed method does not model Phase I,so it is treated as it is, however, if any region is in this phase, it is assumed to be in Phase II in the following period, so this first phase is not relevant for the projections.

#### 3.2.1 Phase II model: Fertility transition

The fertility transition phase is modelled by a random process with drift specified as

$$\begin{array}{c} f_{c,t+1}=f_{c,t}-d_{c,t}+\varepsilon_{c,t},\;\text{for}\;\tau_{c}\leq t<\lambda_{c} \end{array}$$ (1)

where *f*_*c*,*t*_ is the TFR over the five-year period *t* in the region *c*, *d*_*c*,*t*_ is the decremental term that models the systematic decline during the fertility transition, *d*_*c*,*t*_ is the decremental term that models the systematic decline during the fertility transition, *varepsilon*_*c*,*t*_ is a random distortion that models the deviation from systematic decline, *τ*_*c*_ is the period of onset of fertility decline and λ_*c*_ is the period of onset of post-transitional Phase III defined above. The distributions of the random distortions in each period are given by

$$\varepsilon_{c,t}∼\{\begin{array}{c} N\left(m_{t},s_{t}^{2}\right)\;\text{for}\;t=\tau_{c}\\ N(0,\sigma {\left(f_{c,t}\right)}^{2})\;\text{otherwise} \end{array}$$

where *m*_*τ*_ is the mean and *s*_*τ*_ is the standard deviation of the distortion in the starting period. The quantity *σ*(*f*_*c*,*t*_) is the standard deviation of the distortions during the subsequent periods, given by the expression

$$\sigma \left(f_{c,t}\right)=c_{1975}\left(t\right)(\sigma_{0}+\left(f_{c,t}-S\right)\left(-aI_{\left[S,\infty \right)}\left(f_{c,t}\right)+bI_{\left[0,S\right)}\left(f_{c,t}\right)\right))$$

where *σ*_0_ is the maximum standard deviation of the distortions, reached at the *S* level of the TFR, and *a* and *b* are standard deviation multipliers to model the linear decrease for larger and smaller TFR results. The constant *c*_1975_(*t*) is added to model the higher error variance of the distortions before 1975 and is given by

$$c_{1975}\left(t\right)=\{\begin{array}{c} c_{1975},\;t\in \left[1950-1955,1970-1975\right]\\ 1,\;t\in [1975-1980,\infty ) \end{array}$$

**Fig 5:**
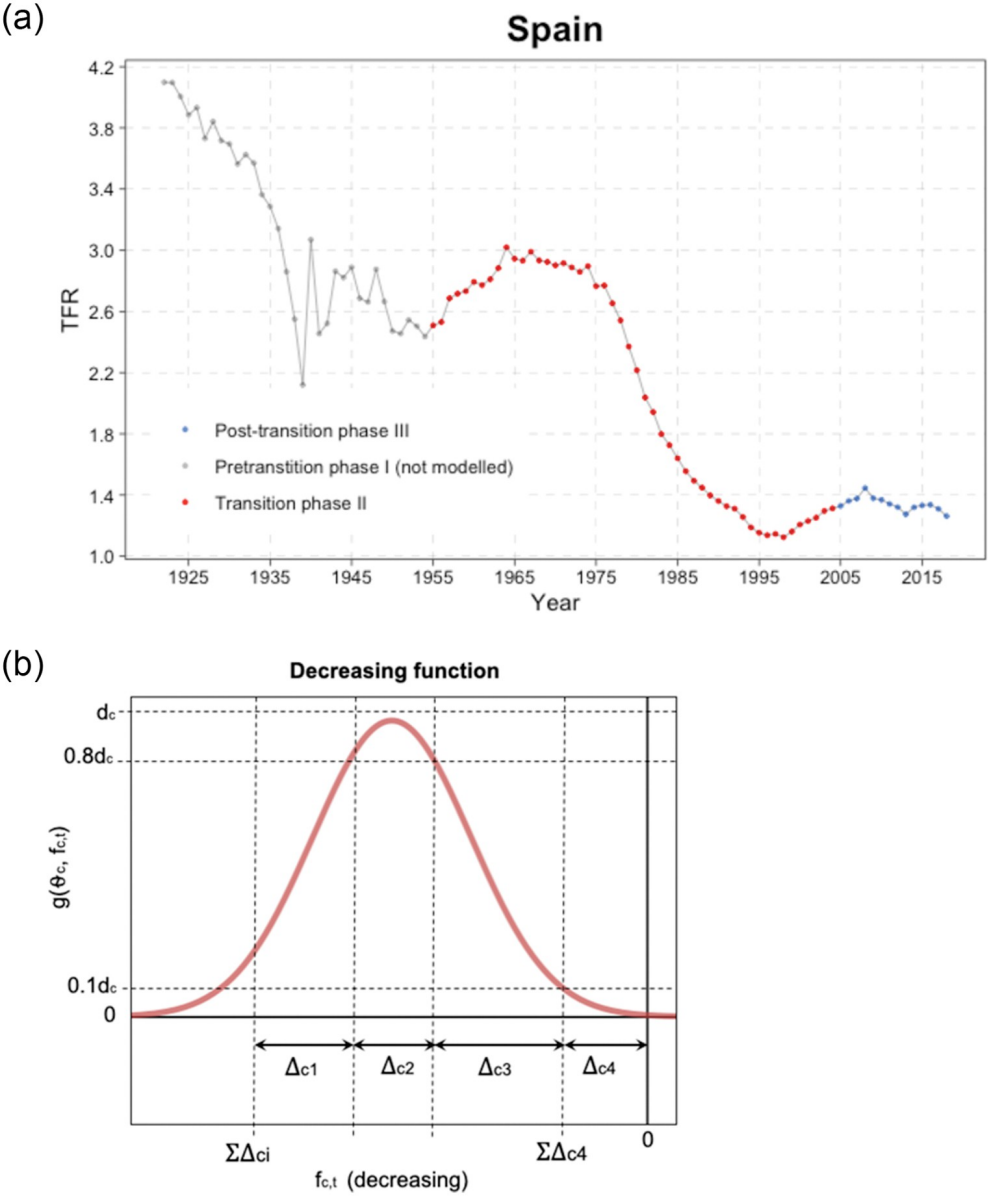
Phases of the TFR evolution. Three phases of the typical TFR evolution for the example of Spain (left); chart of a double logistic decline curve (right). The right figure shows a double logistic decline curve for Spain with its parameters defining the shape *f*_*c*,*t*_ on the *x* axis denotes the TFR, while *g*(*θ*_*c*_, *f*_*c*,*t*_) on the *y* axis denotes the first order difference in TFR. *Source:* Own elaboration.

The decrement *d*_*c*,*t*_ in (1) is modelled as a function of the level of the TFR as follows:

$$d_{c,t}=d\left(\theta_{c},\lambda_{c},\tau_{c},f_{c,t}\right)=\{\begin{array}{cc} g(\theta_{c},f_{c,t}) & \text{for}\;f_{c,t}>1\\ 0 & \text{otherwise} \end{array}$$

where *g*(⋅, ⋅) is a parametric declining function. This function specifies a five-year decline (decrement) as a function of the normal level of the TFR and the vector*θ*. The decrement function is the sum of two logistic functions, i.e. a double or bi-logistic function (as detailed in United Nations, Dept. of Social and Economic Affairs, Population Division, 2019 [94]). The double logistic function with the region-specific vector parameter *θ* = (△_*c*1_, △_*c*2_, △_*c*3_, △_*c*4_, *d*_*c*_) is given by

$$\frac{-d_{c}}{1+\text{exp}(-\frac{2\text{ln}(p_{1})}{{△}_{c1}}\left(f_{c,t}-\sum_{i}{△}_{ci}+0.5{△}_{c1}\right))}+\frac{d_{c}}{1+\text{exp}(-\frac{2\text{ln}(p_{2})}{{△}_{c3}}\left(f_{c,t}-{△}_{c4}+0.5{△}_{c3}\right))}$$

where *d*_*c*_ is the maximum possible rate of decline, *p*_1_ = *p*_2_ = 9 are constants and ${△}_{ci}^{\prime }s$ describe the TFR ranges within which the rate of fertility decline changes, where $U_{c}=\sum_{i=1}^{4}{△}_{ci}$ is the level of onset of fertility decline (Fig 5).

The parameters of the declining function are estimated for each region. For regions in which the onset period *U*_*c*_ of the fertility transition is within the observation period, the onset level *U*_*c*_ is set to the TFR in that period, $U_{c}=f_{c,\tau_{c}}$. For regions where the transition started before the observation period, the onset level is added as a parameter to the model, with the prior distribution

$$U_{c}∼\left(\text{min}\left\{5.5,\underset{t}{\text{max}}f_{c,t}\right\},8.8\right).$$

Given the starting level *U*_*c*_, the five parameters that determine the rate of fertility decline and the time it takes for the transition in region *c* are △_*c*4_, {△_*ci*_/(*U*_*c*_−△_*c*4_):*i* = 1, 2, 3}, and *d*_*c*_.

To estimate the parameters in each region, we use a Bayesian hierarchical model [95, 96] given by

$$\begin{array}{cc} d_{c}^{*} & =\text{log}(\frac{d_{c}-0.25}{2.5-d_{c}}),\\ d_{c}^{*} & ∼N(\chi ,\psi^{2}),\\ {△}_{c4}^{*} & =\text{log}(\frac{{△}_{c4}-1}{2.5-{△}_{c4}}),\\ {△}_{c4}^{*} & ∼N({△}_{4},\delta_{4}^{2}),\\ p_{ci} & =\frac{{△}_{ci}}{U_{c}-{△}_{c4}}\text{para}\;i=1,2,3,\\ p_{ci} & =\frac{\text{exp}(\gamma_{ci})}{\sum_{j=1}^{3}\text{exp}\left(\gamma_{cj}\right)},\\ \gamma_{ci} & ∼N(\alpha_{i},\delta_{i}^{2}), \end{array}$$

with parameters of mean and variance {*χ*, *ψ*^2^, △_4_, *δ*_4_, ***α***, ***δ***}.

In the post-transition modelling phase the change in TFR is modelled by a first-order autoregressive time series model, i.e. AR(1) with a mean set approximately at the replacement fertility level *μ* = 2.1:

$$f_{c,t+1}∼N\left(\mu +\rho \left(f_{c,t}-\mu \right),s^{2}\right)\;\text{for}\;t\leq \lambda_{c}$$

where *ρ* is the autoregressive parameter with |*ρ*|<1 and *s* is the standard deviation of the random errors. Both parameters are estimated by maximum likelihood.

Finally, the projections of the TFR during the fertility transition for regions in *Phase II* are based on the model for this phase, as discussed above, using the sample posterior distribution of the model parameters.

Finally, according to [27] is important to remark that the median (rather than the mean) is used as the best ‘projection’ because of its robustness to the tail behaviour of the posterior distributions: regardless of the shape of the posterior distribution, half of the TFR trajectories are above, and half of the trajectories are below the median.

## 4 Results

### 4.1 Fitting and projections at national level

The probabilistic projections for the Spanish TFR at regional levels include uncertainty bounds. These bounds are remarkable for less developed regions, with less available data or where more recent observed data entries do not exist.

From a computational point of view, to obtain these projections, three steps have been followed in the following order:

1. Adjustment of the TFR projection model, for which:
  - The starting period of *Phase II* and the starting period of *Phase III* have been calculated for each region (*τ*_*c*_ and λ_*c*_). This phase, in our case, is common to the whole process.
  - A posterior sample of the *Phase II* model parameters has been obtained using the MCMC algorithm.
2. Future TFR trajectories have been generated (this step includes the estimation of the AR(1) model parameters in *Phase III* using the maximum likelihood estimation method).
3. The results have been analysed using a set of functions that summarise, plot, diagnose and export the results of the previous two steps.

We detail the logical sequence of execution for step 1, i.e., the TFR projection model fitting in the S1 Appendix.

A summary of the simulated parameters for Spain are shown in Tables 1 and 2: and the statistical parameters for the projected trajectories are shown in Table 3:

**Table 1:** Empirical mean and standard deviation for each variable, plus standard error of the mean.

	Mean	SD	Naive SE	Time-series SE
*d*_1_	0.87805	0.222604	0.0040642	0.0242830
*d*_2_	0.93463	0.290494	0.0053037	0.0444689
*d*_3_	0.85959	0.205225	0.0037469	0.0243039
${△}_{c4}^{*}$	0.49771	0.312075	0.0056977	0.0266451
*d*_4_	1.18525	0.657369	0.0120019	0.1198672
*U*_*c*724_	7.23997	0.942422	0.0172062	0.0172611
*d*_*c*724_	0.15115	0.057671	0.0010529	0.0015251
△_*c*4, *c*724_	1.77613	0.343262	0.0062671	0.0105829
*γ*_*t*1, *c*724_	0.06726	0.069615	0.0012710	0.0018478

**Table 2:** Quantiles for each variable.

	2.5%	25%	50%	75%	97.5%
*d*_1_	0.50418	0.71747	0.86230	1.01957	1.34928
*d*_2_	0.47444	0.73158	0.90539	1.08324	1.65377
*d*_3_	0.48672	0.71673	0.85140	0.98991	1.29804
${△}_{c4}^{*}$	-0.09077	0.31455	0.51002	0.69595	1.04759
*d*_4_	0.54755	0.85690	1.06003	1.29358	3.32724

**Table 3:** Projected TFR simulations for Spain.

Year	Mean	SD	2.5%	5%	10%	25%	50%	75%	90%	95%	97.5%
2023	1.38	0.102	1.16	1.19	1.24	1.33	1.39	1.45	1.49	1.54	1.55
2028	1.41	0.128	1.14	1.18	1.25	1.34	1.41	1.51	1.57	1.58	1.61
2033	1.46	0.152	1.17	1.19	1.26	1.38	1.47	1.56	1.64	1.68	1.70
2038	1.52	0.179	1.18	1.20	1.26	1.39	1.54	1.63	1.72	1.78	1.84
2043	1.55	0.198	1.17	1.19	1.29	1.39	1.56	1.69	1.79	1.83	1.84
2048	1.55	0.185	1.22	1.22	1.29	1.41	1.58	1.67	1.77	1.84	1.85
2053	1.58	0.181	1.22	1.26	1.37	1.45	1.59	1.72	1.78	1.87	1.90
2058	1.61	0.185	1.25	1.31	1.40	1.48	1.61	1.75	1.85	1.91	1.97
2063	1.63	0.198	1.23	1.28	1.39	1.50	1.64	1.78	1.84	1.96	2.00
2068	1.64	0.192	1.27	1.33	1.35	1.52	1.65	1.78	1.86	1.90	2.02
2073	1.65	0.173	1.28	1.32	1.43	1.54	1.68	1.78	1.82	1.89	1.94
2078	1.67	0.188	1.32	1.37	1.41	1.55	1.70	1.81	1.86	1.98	2.01
2083	1.67	0.194	1.31	1.32	1.37	1.53	1.71	1.83	1.87	1.92	1.96
2088	1.69	0.189	1.35	1.40	1.45	1.55	1.70	1.82	1.89	1.99	2.07
2093	1.70	0.201	1.41	1.42	1.47	1.54	1.70	1.85	1.93	1.99	2.11
2098	1.70	0.220	1.37	1.43	1.46	1.53	1.67	1.87	1.96	2.00	2.15

**Fig 6:**
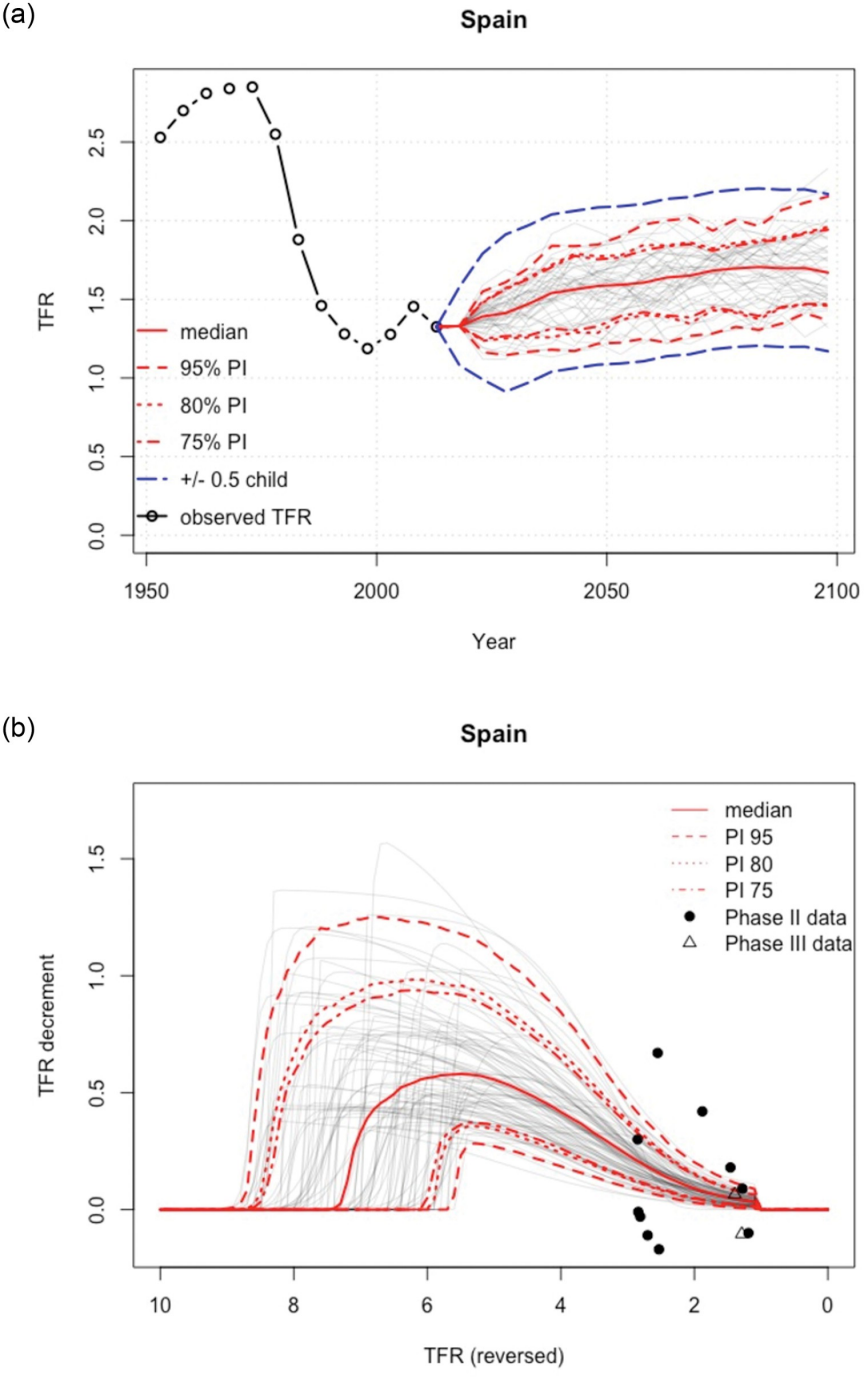
Probabilistic projections of TFR. TFR projections: median, 80%, and 95% prediction intervals and high/low fertility variant (left); Probabilistic projections of total fertility. Decline curves (based on the double logistic function) from the Bayesian hierarchical model, Median, 80% and 95% prediction intervals (right). *Source:* own elaboration.

The projected simulations of the TFR are shown in Fig 6:

The left figure shows the Bayesian hierarchical model projections of total fertility using the estimates from the *‘World Population Prospects. Revision 2019’* [94]. Note that only a small selection of the probabilistic total fertility trajectories (grey lines) is shown for illustration. The median projection is the bold solid red line, and the 80% and 95% projection intervals are shown as dashed and dotted red lines, respectively. High-low fertility variants correspond to +/- 0.5 children around the median trajectory shown as blue dashed lines. The replacement level of children per woman would be at level 2.1, although it is not shown in the graph.

The graph on the right shows the Bayesian Hierarchical Model (BHM) decline curves for total fertility that have been made also with the fertility estimates from the *‘World Population Prospects. Revision 2019’*. As in the previous model, it should be noted that only a small selection of the double logistic probability trajectories (grey lines) is shown for illustration. The observed five-year decreases in total fertility level are shown with black dots if they refer to periods of the fertility transition defined as *Phase II* (i.e. from high to low fertility). The median projection is the bold solid red line, and the 80% and 95% projection ranges are shown as dashed and dotted red lines, respectively. *Phase I* data refer to the period before the onset of the fertility transition (if it occurred since 1950). *Phase III* data refer to the period after the low-fertility transition not modelled using the double logit model, but using a first-order autoregressive time series model, AR(1).

The fit of the model simulation for Spain is quite accurate as the starting point puts the fertility rate at around 1.3 births per woman, similar to that provided by Eurostat which is 1.23 (https://ec.europa.eu/eurostat). In addition to this, the most interesting insight is the projections made by the model, which can be seen to be approaching the rate of 1.8 births per woman, a rate that is in line with the projections made by Osés-Arranz et al., (2018) [77] discussed earlier when we talked about the projection models for the Spanish case.

### 4.2 Projections at the regional level

**Fig 7:**
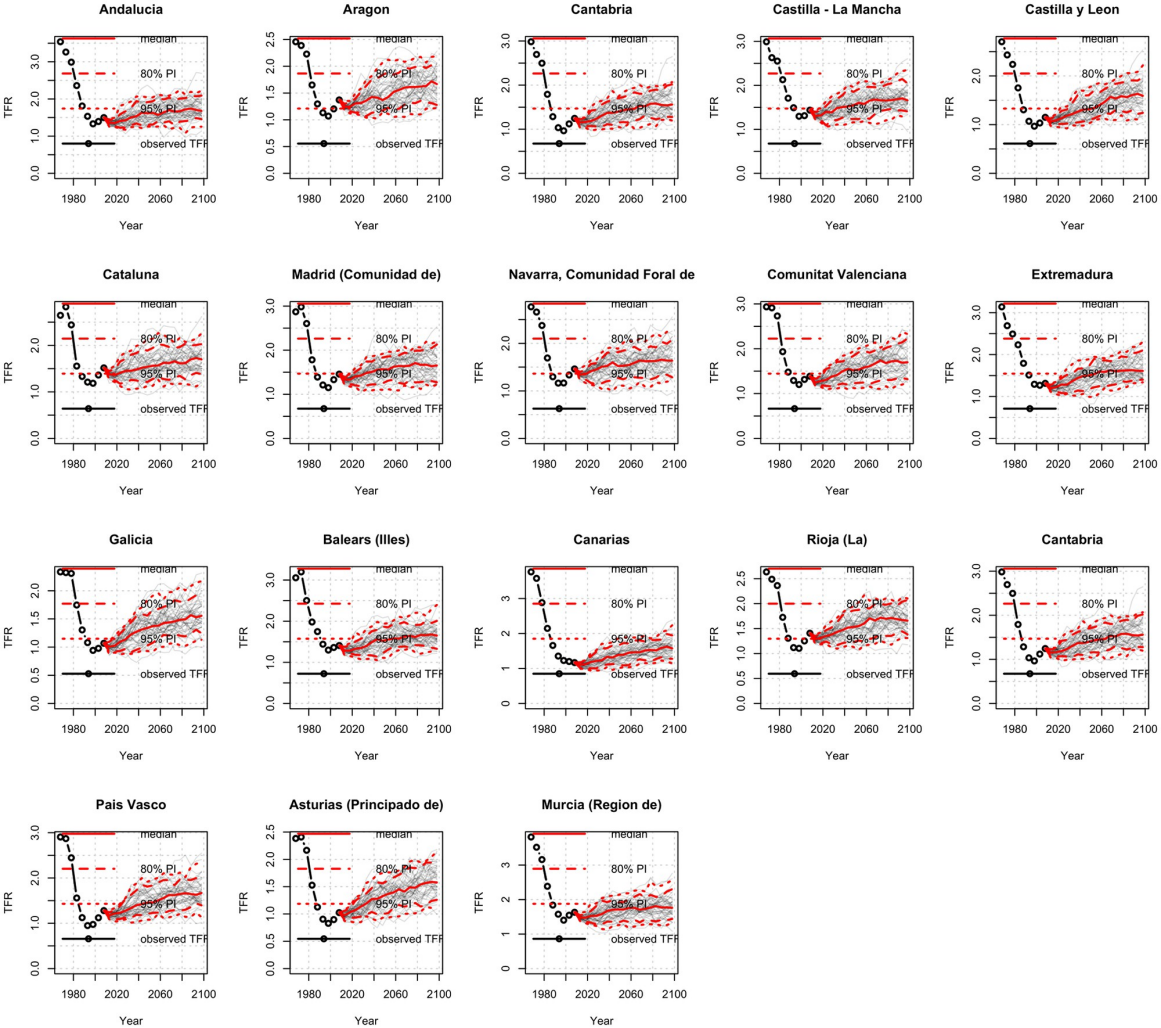
Probabilistic projections of the TFR at the regional level. TFR projections: median, 80%, and 95% prediction intervals and high/low fertility variant for each region. *Source:* own elaboration.

One of the main contributions of this work has been the fitting and projection of the TFR at the regional level. We have followed the same methodology and procedure as for the national case and computed the probabilistic projections for the regional TFR including also uncertainty bounds. Fig 7 shows the TFR for each region (Spanish regional division accounts for a total of 17 regions or *‘Comunidades Autónomas’*) each of them with a certain degree of autonomy but dependent on the national government itself.

#### 4.2.1 Discussion on regional differences

Spanish national-level projections are included in the upper left chart just to compare with the regions. Although the projected trend in TFR is unequivocally upward in the long-term for all Spanish regions, the intensity and timing are not the same for all of them. There are regions which, on the one hand, showed greater precocity in the onset of the fertility decline in the past, so it is worth highlighting these peculiarities; on the other hand, it is not possible to speak of uniformity concerning the amount of the increase, since this is not homogeneous throughout the territory and, likewise, not all of them always coincide in the changes in the trend. To compare the evolution of each region with that of Spain as a whole, the provinces have been grouped by area, making them coincide with their membership in the current autonomous communities (the so-called regions).

Particularly interesting are the cases of those regions with a projected TFR close to the replacement threshold level, that is 2–2.1 births, i. e. Cataluña, Andalucía, Canarias and Murcia, especially because as a starting point they are quite below the threshold, in any case, these are the regions that contribute most to the increase in the TFR at the national level and although the Spanish population is projected to grow for the rest of the century, it tends to stabilize at the end. For these regions mentioned, immigration plays an important role as these regions concentrate higher rates of people coming from other countries, especially Andalucía, Canarias and Murcia are receiving people from African countries cities like Almería, Melilla, Ceuta, La Línea de la Concepción, Algeciras, Sanlúcar de Barrameda (Andalucía); Lorca and Cartagena (Murcia); Santa Coloma de Gramenet, Rubí, Reus, Sabadell and Terrassa (Cataluña) are the cities with the highest number of children per woman [97]. The migration phenomenon is relevant in the Spanish case and although our analysis has been carried out without differentiating the nationality the migration issue remains open for further research to analyze the fertility pattern between nationalities (Spanish and foreigners).

On the other hand, the regions with lower projected TFR were Cantabria, Castilla-León, Extremadura, and Galicia; in fact, cities like Torrelavega, Santander, Gijón (Cantabria); Ferrol, Vigo and A Coruña (Galicia) are the cities with the lowest number of children per woman [97] this is somehow related with another important problem faced by Spain: the depopulation of rural areas. According to data from 2020 [98], the five Spanish functional urban areas with the largest populations (over one million inhabitants) are Madrid (6.30), Barcelona (5.22), Valencia (1.58), Seville (1.31) and Málaga (1.01). Together, they account for 15.42 million inhabitants, 32.56% of the Spanish population. That means that a significant part of the rural population in areas with fewer employment options has tended to leave their homeland, mainly for the cities that make up the major national economic centres or to go abroad. This is in line with the TFR at the municipality level map shown in Fig 3 where the areas with a TFR close to zero are placed in Extremadura, Cantabria and Castilla-León. They are regions with lower rates of employment, industrialization and population. Note that Seville and Málaga are urban areas with over one million inhabitants. Despite Andalucía having small areas suffering depopulation, there is a concentration in the largest cities, contributing, as we see above to increase the fertility in this region.

## 5 Conclusions

In this study TFR evolution and probabilistic projections for Spain have been implemented and analyzed; in the research section, we conducted a review of the literature on fertility and age structure models which has tried to collect those works that have contributed to the improvement in this demographic area. In the empirical section, we used a Bayesian hierarchical model to fit and project the TFR at the regional level.

Fertility decline is a phenomenon that has traditionally been of interest to demographers, economists, policy-makers, politicians and, more recently, the general public. Population problems mainly refer to the progressive ageing and non-replacement of generations in developed countries as a result of the fertility decline.

In Spain, we have seen that two effects play a crucial role: migration and depopulation but in addition to that, the lack of public aid, the loss of income for women after motherhood, the difficulty of reconciling working time and parental (and due to this, the lack of co-responsibility of many men in child-bearing) are among the worrying factors of the phenomenon. It is a structural problem because demographic factors themselves are not strong enough to push fertility down, but an exogenous factor, industrialization, is needed to cause a profound change in the social, economic and cultural structures of a country and force the demographic system as a whole to adapt so that the demographic transition can begin, [99].

The probabilistic model we have used, adapted to the Spanish population and used to simulate projections tens of years ahead, is a very useful tool to measure and manage longevity risk. This is an important issue for those who wish to hedge longevity risk using published mortality rates, be they governments, pension plans, insurers or banks. Moreover, longevity-based risk, while a challenge not only for demographers, statisticians and actuaries but also for government agencies, can also present a broader problem for insurance companies that use external data, such as population data, rather than their internal policy data in their reserving models. The need to quantify and reserve for any potential basis risk is receiving increasing focus, particularly under Solvency II, therefore given the continuing increases in life expectancy, the use of the best available methods is now mandatory, particularly by actuaries practising in the insurance and financial risk industry, where the ramifications of inaccurate forecasts are acute and although the magnitudes of ageing are uncertain, and forecast errors are likely to be large, ageing policies can anticipate this, uncertainty should not imply inaction and forecasts contain information that can be used in the design of social policies that will have an impact on both the present and, more importantly, the future population.

However, probabilistic models based on Bayesian methods and hence the one we used here, have some limitations. First, as the Bayesian inference entails the choice of prior probabilities, the construction of hierarchical models relies on certain assumptions that results are subject to such assumptions for countries with no or limited data. In our case, this was not a problem as we had enough data but for countries more limited, before fitting the model, data are adjusted for *Completeness of Birth Reporting* (provided by the UNPD) and the biases used WPP 2019 (World Population Prospects) estimates as reference levels [100]. In addition to that, from a computational point of view is an intensive process, especially for models involving many variables. We ran simulations for countries with large datasets with many variables being estimated, and it took time.

Fertility models are used in a wide variety of situations, for example, to smooth observed data as inputs into population projections or other analytical exercises. It is important to note that the accuracy of models predicting demographic phenomena is important when judging the quality of population forecasts. In aspects such as information content, for example, one might ask whether the forecast predicts only the total population or also age groups. For policy purposes, on the other hand, it is relevant to discern whether the predicted trend implies immediate policy measures. However, the degree to which the forecast reflects actual trends is a key factor in assessing its quality, particularly when the forecast is used for planning purposes. For example, imagine a forecast for which the odds are one against two that will cover the actual trends. Such a forecast must be handled much more cautiously than one that can be expected to be wrong only one in five times.

Predictions of demographic phenomena (not only fertility but mortality and migration) have become increasingly important in recent decades as life expectancy has increased rapidly. Over the years, a large number of different approaches have been proposed to model these phenomena, ranging from the simplest (the UN model programme) to the most complicated (the Coale-Trussell model, for example).

Studies have shown that a three-parameter model can capture most of the variation in observed fertility patterns. Models with more parameters, for most purposes, are not necessary, and difficulties may be experienced in fitting such models to a small number of data. The Gompertz relational model, for instance, is a very flexible three-parameter system for modelling fertility, which has found application in many areas of demographic work.

## Supporting information

S1 Appendix Sequence of the adjustment of the TFR projection model. Simulations of MCMC parameters and density distribution functions.
(ZIP)
