# Supplementary material for: Projecting Spanish fertility at regional level: A hierarchical Bayesian approach
Source: PLoS One. 2022 Oct 18;17(10):e0275492. doi: 10.1371/journal.pone.0275492 (PMC9578621; doi:10.1371/journal.pone.0275492)
Supplement: S1 Appendix — Simulations of MCMC parameters and density distribution functions. (ZIP) [file pone.0275492.s001.zip › s1.pdf]

Supporting information

S1 Appendix. Sequence of the adjustment of the TFR projection model and hyperparameters computation. The first code line creates a directory where all the simulations are stored, from there, the adjustment of the TFR projection takes place using the function `run.tfr.mcmc`. Depending on the size of the MCMC sample, steps 1(b) and 2 are quite long in terms of execution, in our case, the expression `nr.chains` determines the number of MCMC chains to simulate and since the aim is to run the model on a realistic basis, we simulated five chains of 10 000 iterations each, additionally, we simulated another 1 000 iterations, making a total of 11 000 iterations, which took us about six hours. All these results were stored in the directory created for this purpose.

```
> simulation.dir <- file.path(getwd(), "fertsimul")
> m1 <- run.tfr.mcmc(nr.chains = 5, iter = 10000, + output.dir = simulation.dir)
> m1 <- run.tfr.mcmc(nr.chains=5, iter=10000, output.dir=simulation.dir, seed=1)
> m2 <- continue.tfr.mcmc(iter=1000, output.dir=simulation.dir)
```

Once the simulations are created, we proceeded to create the trajectories with the following function:

```
> pred1 <- tfr.predict(sim.dir = simulation.dir, end.year = 2100, burnin = 2000,
+ nr.traj = 3000, verbose = TRUE)
```

Where 2000 simulations at the beginning of each chain will be eliminated and 3000 parameter values from the remaining 5 chains of 9000 simulations each will be used, i.e. 45000 iterations to generate the trajectories. To summarise, using the command `summary`:

```
> summary(m3, meta.only = TRUE)

MCMC parameters estimated for 196 countries.
Hyperparameters estimated using 196 countries.

WPP: 2019
Input data:  TFR for period 1950 - 2019.

Number of chains = 5
Iterations = 1 :  55000
Thinning interval = 1
Chains sample sizes:  11000, 11000, 11000, 11000, 11000
```

Where we see that the data simulated with MCMC and the hyperparameters $\{\chi, \psi^2, \triangle_4, \alpha_i, \delta_i, \delta_4\}$ are for 196 countries.

Focusing on Spain, with the following commands we will obtain all the relative and necessary information for this country:

```
> summary(m3, country = "Spain", par.names = NULL, thin = 10, burnin = 2000)
```

which, as an extract, is shown in the following output obtained from the simulation:

```
Country:  Spain

Iterations = 2010:11000
Thinning interval = 10
Number of chains = 5
Sample size per chain = 600

1.  Empirical mean and standard deviation for each variable, plus standard error
of the mean:
                        Mean       SD      Naive SE   Time-series SE
      delta1         0.87805  0.222604  0.0040642     0.0242830
      delta2         0.93463  0.290494  0.0053037     0.0444689
      delta3         0.85959  0.205225  0.0037469     0.0243039
    Triangle4        0.49771  0.312075  0.0056977     0.0266451
      delta4         1.18525  0.657369  0.0120019     0.1198672
      U_c724         7.23997  0.942422  0.0172062     0.0172611
      d_c724         0.15115  0.057671  0.0010529     0.0015251
 Triangle_c4_c724    1.77613  0.343262  0.0062671     0.0105829
   gammat_1_c724     0.06726  0.069615  0.0012710     0.0018478
        ...

2.  Quantiles for each variable:

              2.5%      25%      50%      75%     97.5%
   delta1    0.50418  0.71747  0.86230  1.01957  1.34928
   delta2    0.47444  0.73158  0.90539  1.08324  1.65377
   delta3    0.48672  0.71673  0.85140  0.98991  1.29804
 Triangle4  -0.09077  0.31455  0.51002  0.69595  1.04759
   delta4    0.54755  0.85690  1.06003  1.29358  3.32724
    ...
```

and using the function **pred2**, we can obtain the statistics for the projected trajectories:

```
> summary(pred2, country = "Spain")
> summary(pred2, country = "Spain")
Country:  Spain

Projections:  17 ( 2018 - 2098)
Trajectories:  3000
Phase II burnin:  2000
Phase II thin:  10
Parameters of AR(1):
mu rho sigma
2.1 0.886 0.102

Projected TFR:

       Mean    SD    2.5%   5%    10%   25%   50%   75%   90%   95%  97.5%
 2013  1.33  0.000  1.33  1.33  1.33  1.33  1.33  1.33  1.33  1.33  1.33
 2018  1.41  0.102  1.21  1.24  1.28  1.34  1.41  1.48  1.54  1.58  1.61
 2023  1.49  0.135  1.21  1.26  1.31  1.40  1.49  1.58  1.66  1.71  1.75
 2028  1.56  0.157  1.25  1.30  1.36  1.45  1.56  1.67  1.77  1.81  1.86
 2033  1.62  0.171  1.29  1.33  1.40  1.51  1.62  1.74  1.84  1.91  1.96
 2038  1.68  0.183  1.32  1.38  1.44  1.56  1.68  1.80  1.92  1.98  2.04
  ...
```

Graphical representation (**fig. 1**) shows the following simulations:

[**Figures S1a and S1b**]

Fig 1. Probabilistic projections of total fertility. Total fertility projections: median, 80%, and 95% prediction intervals and high/low fertility variant (left); Probabilistic projections of total fertility. Decline curves (based on the double logistic function) from the Bayesian hierarchical model, Median, 80% and 95% prediction intervals (right). Source: Authors' elaboration.

The simulation of the iterations for the hyperparameters $\{\chi, \psi^2, \triangle_4, \alpha_i, \delta_i, \delta_4\}$ can also be analysed and explored graphically using two functions of the package: one to visualise the country independent parameters, and the other to visualise the country-specific parameters, which, in **R**, is given by the command

```
> tfr.partraces.plot(mcmc.list = m3, par.names = "Triangle4", nr.points = 100)
```

for the figure 2, $\triangle_4$, (left) containing one trace for each MCMC chain (in our example 5 traces).

[**Figures S2a and S2b**]

Fig 2. Parameters' simulation. MCMC parameters' simulation for $\triangle_4$ (left). Spain's parameters' simulation, $\triangle_{c4}$ (right). Source: Authors' elaboration.

and for the command

```
> tfr.partraces.cs.plot(country = "Spain", mcmc.list = m3, nr.points = 100,
+     par.names = "Triangle_c4")
```

for the right figure $\triangle_{c4}$ specific to Spain.

It is also possible to visualize the distributions of the MCMC hyperparameters (fig.**??**), and whose equivalence with the code in **R** is showed as follows:

χ	ψ	$\triangle_4$	$\alpha_{1,2,3}$	$\delta_{1,2,3}$	δ_4	$\frac{exp(\alpha_i)}{\sum_j exp(\alpha_i)}$	a	b	S
chi	psi	Triangle4	alpha	delta	delta4	alphat_i	a_sd	b_sd	S_sd

σ_0	c_{1975}	m_τ	s_τ
sigma0	cons_sd	mean_eps_tau	sd_eps_tau

The script used is given by the function:

```
> tfr.pardensity.plot(pred2, par.names = c(‘‘alphat", "Triangle4", "delta",
+     "sigma0"), dev.ncol = 4, bw = 0.05)
```

[**Figure S3**]

Fig 3. Density of distributions of various country-independent parameters. Source: Authors' elaboration.
