# Supplementary figures and images for: Projecting Spanish fertility at regional level: A hierarchical Bayesian approach
Source: PLoS One. 2022 Oct 18;17(10):e0275492. doi: 10.1371/journal.pone.0275492 (PMC9578621; doi:10.1371/journal.pone.0275492)

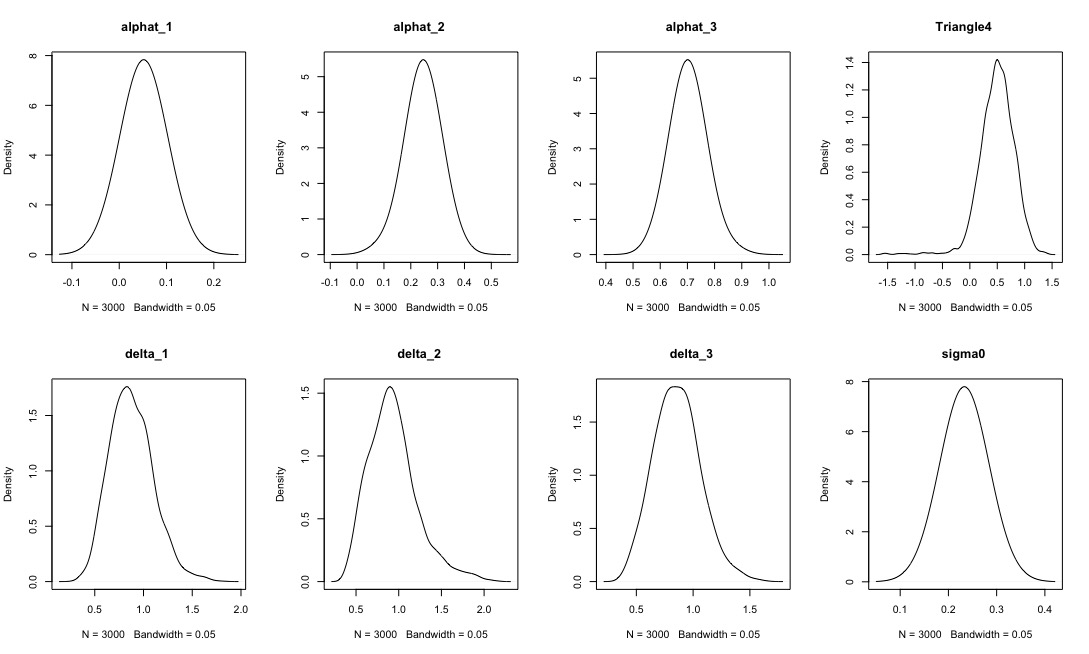

Supplement: S1 Appendix — Simulations of MCMC parameters and density distribution functions. (ZIP) [file pone.0275492.s001.zip › s3.tif]

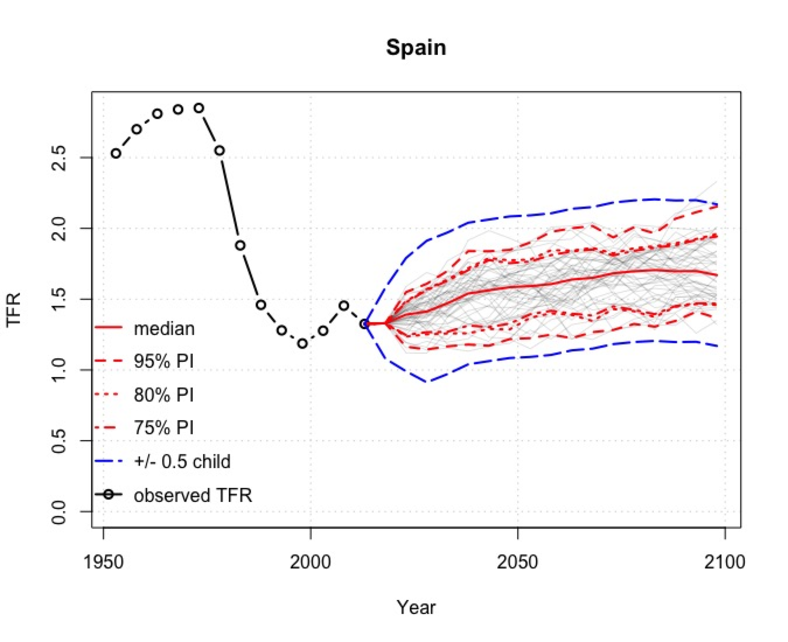

Supplement: S1 Appendix — Simulations of MCMC parameters and density distribution functions. (ZIP) [file pone.0275492.s001.zip › s1a.tif]

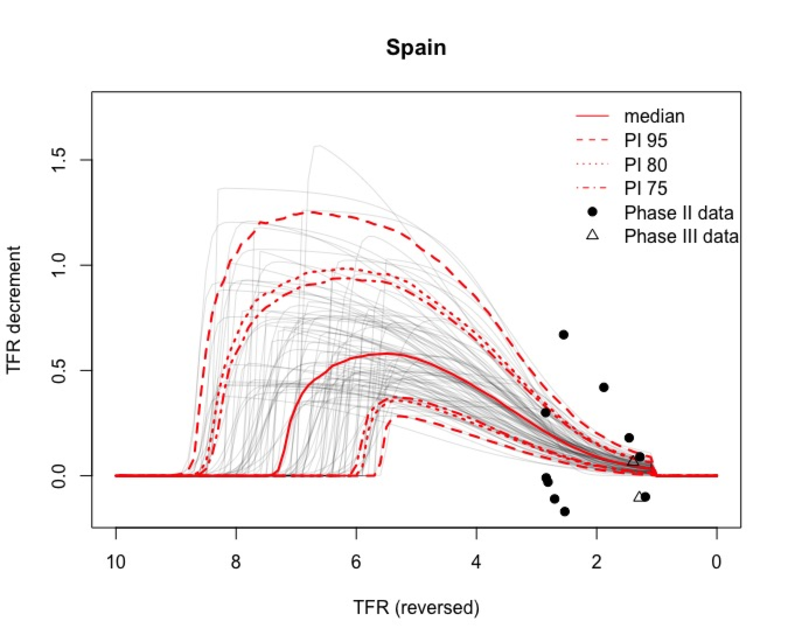

Supplement: S1 Appendix — Simulations of MCMC parameters and density distribution functions. (ZIP) [file pone.0275492.s001.zip › s1b.tif]

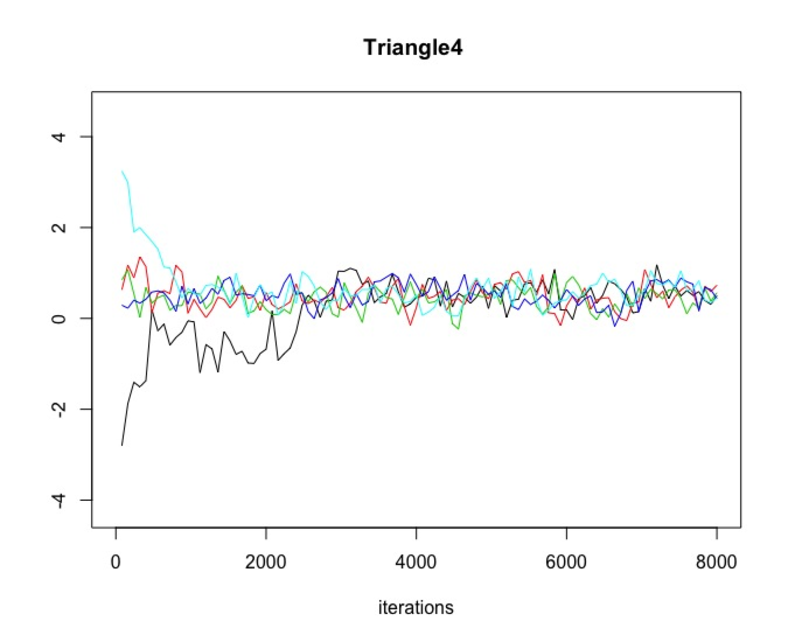

Supplement: S1 Appendix — Simulations of MCMC parameters and density distribution functions. (ZIP) [file pone.0275492.s001.zip › s2a.tif]

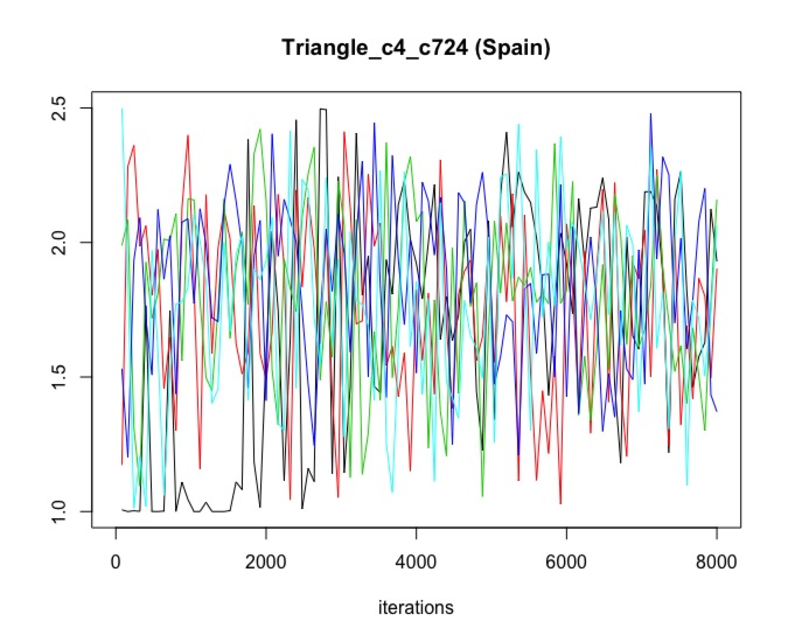

Supplement: S1 Appendix — Simulations of MCMC parameters and density distribution functions. (ZIP) [file pone.0275492.s001.zip › s2b.tif]
